# Prognostic Indicators of Carbapenem‐Resistant *Acinetobacter baumannii* Infection: A Meta‐Analysis and Systematic Review

**DOI:** 10.1002/hsr2.71639

**Published:** 2025-12-14

**Authors:** Jie Jin, Yan Hu, Ling Yan Du

**Affiliations:** ^1^ Leshan Vocational and Technical College Leshan City Sichuan Province China; ^2^ Leshan People's Hospital Leshan City Sichuan Province China

**Keywords:** carbapenem‐resistant *Acinetobacter baumannii*, concomitant disease, mortality, prognostic indicators

## Abstract

**Background:**

Carbapenem‐resistant *Acinetobacter baumannii* (CRAB) infections are clinically difficult to treat owing to their lack of effective treatments and high mortality rates. This calls for the development of prognostic indicators to facilitate risk stratification and patient management.

**Aims:**

To identify prognostic indicators of CRAB infections through a systematic review and meta‐analysis.

**Methods:**

A systematic search was performed on PubMed, Embase, and Cochrane Library up to November 2024. Studies that reported the CRAB infections and their adverse outcomes (e.g., mortality, acute kidney injury, recurrent infections) were eligible for enrollment. Data were extracted from the studies by two reviewers, and the quality of the studies was determined using the Newcastle−Ottawa Scale (NOS). Meta‐analysis was performed using random‐effects or fixed‐effects models while considering the heterogeneity determined using the *I*² statistics.

**Results:**

Fourteen studies comprising 2250 patients were enrolled in the study. Notably, cardiovascular disease (OR: 1.59, 95% CI: 1.17–2.17), acute kidney injury (OR: 3.15, 95% CI: 1.69–5.88), shock (OR: 2.33, 95% CI: 1.50–3.63), pneumonia (OR: 1.28, 95% CI: 1.02–1.60), hematologic malignancy (OR: 2.23, 95% CI: 1.55–3.21), neutropenia (OR: 2.74, 95% CI: 1.48–5.06), and thrombocytopenia (OR: 1.49, 95% CI: 1.10–2.02) were found to be significant prognostic indicators predicting poor outcomes. The use of steroid therapy (OR: 1.29, 95% CI: 1.01–1.64) was linked to increased mortality risk.

**Conclusion:**

In this meta‐analysis, we detected several prognostic indicators that correlated with poor adverse outcomes in patients with CRAB infections, which may help clinical decision‐making and risk‐stratification strategies. **Trial Registration:** PROSPERO 2024 CRD42024565429 Available from: https://www.crd.york.ac.uk/prospero/display_record.php?ID=CRD42024565429.

**Abbreviations**
BSI: bloodstream infection
CARES: Chinese Antimicrobial Resistance Surveillance of Nosocomial Infections
CI: confidence interval
CKD: chronic kidney disease
CRAB: carbapenem‐resistant *Acinetobacter baumannii*
ECMO: extracorporeal membrane oxygenation
ES: effect size
HR: hazard ratio
ICU: intensive care unit
IL‐13: interleukin‐13
MDR: multidrug‐resistant
NOS: Newcastle−Ottawa scale
OR: odds ratio
PI: prediction interval
PROSPERO: International Prospective Register of Systematic Reviews
ST: sequence type (e.g., ST2 *Acinetobacter baumannii*)
WHO: World Health Organization
τ²: between‐study variance

## 1 Introduction

Carbapenem‐resistant *Acinetobacter baumannii* (CRAB), one of the six superbugs identified by the World Health Organization, is an intractable clinical challenge globally [1, 2]. It is a major cause of nosocomial infection which is pervasive in hospital environments, with high capacity to evade the host immune responses. CRAB infections are also highly susceptible to antibiotic resistance.

In recent years, research on CRAB infections has increased worldwide, with significant efforts directed toward identifying the mechanisms of drug resistance and developing treatment strategies [3, 4, 5]. So far, several studies have investigated the genomic features of locally prevalent strains [6], and strategies have been developed to improve the efficacy of tigecycline and polymyxin [7, 8, 9]. These studies not only deepen our understanding of CRAB infections but also provide valuable scientific foundations that guide further treatment and control measures.

The pathogenicity of *Acinetobacter baumannii* is primarily driven by its strong antibiotic resistance and rapid acquisition of resistance mechanisms, making it a challenging clinical infection to treat. It is, therefore, crucial to identify and predict factors that influence adverse outcomes following CRAB infections. The nosocomial infection rate of CRAB is higher than 60%, and its clinical outcomes are poor, characterized by high mortality, prolonged hospitalization, and increased medical costs [10]. CRAB infections present with complex clinical manifestations, including severe multi‐organ dysfunction and extended hospital stays [11, 12, 13, 14]. Several risk factors for *A. baumannii* infections have been reported, including prolonged hospitalization, ICU stays, mechanical ventilation, invasive procedures, exposure to antibiotics, and severe underlying diseases [15, 16, 17]. Therefore, timely prediction of these adverse outcomes may help to develop personalized treatment plans.

Numerous studies have developed prediction tools for clinical outcomes following CRAB infections by analyzing clinical data, laboratory indicators, and infection control measures. The commonly used clinical factors include patient age, ICU admission, comorbidities (e.g., chronic liver disease, chronic kidney disease [CKD], cardiovascular disease), incidence of pneumonia, and other secondary conditions [18, 19, 20]. However, due to variations in study design, sample size, data collection methods, and analysis techniques, it is often challenging to obtain robust conclusions [21]. Moreover, the epidemiological features of CRAB infections vary regionally, which complicates the formulation of uniform treatment guidelines. In addition, the varied definitions and evaluation methods for prognostic predictors across studies further complicate the integration of findings from such investigations. Therefore, systematic reviews and meta‐analyses are urgently needed to synthesize existing data and provide more reliable evidence for prognostic assessment of CRAB infections.

Considering the significant negative impact of CRAB infections and the limitations of existing studies, this systematic review and meta‐analysis aim to address this knowledge gap by comprehensively analyzing prognostic indicators of CRAB infection outcomes. The primary objective of this study is to identify prognostic indicators of CRAB infections through a meta‐analysis. Relevant literature was searched, and studies that met specific criteria, especially those reporting adverse outcomes in patients with CRAB infections, were explored. The Newcastle‐Ottawa Scale (NOS) was employed to investigate the quality of the enrolled studies [22]. Furthermore, the basic characteristics, clinical information, and their association with patient prognosis were analyzed. The aim of the study is to generate a risk stratification tool, which can help clinicians and public health policymakers to formulate strategies for reducing the global burden of CRAB infections.

## 2 Methods

### 2.1 Search Strategy

Two independent investigators (J.J. and Y.H.) searched the PubMed, the Cochrane Library databases, and Web of Science from the inception to November 2024. The key search terms used are shown in the Table S1 and included: (Resistance OR resistant OR “drug resistance” OR “multiple drug resistance” OR “extensively drug resistance”) AND (Carbapenem OR carbapenems OR “carbapenem antibiotics”) AND (*A. baumannii* OR “hospital‐acquired infections” OR “severe infections” OR “bloodstream infection” OR “urinary tract infection” OR “wound infections”) AND (Prognosis OR prognostic OR “risk factor” OR death OR hospitalized OR readmission OR “disease outcome” OR “survival rate” OR survival OR “cure rate” OR cure OR “disease resolution” OR “risk assessment” OR “prognostic models” OR “prediction models”). In addition, the investigators searched the reference lists of the identified studies to identify additional relevant articles. Only studies published in English were enrolled. This systematic review and meta‐analysis were conducted in accordance with the Preferred Reporting Items for Systematic Reviews and Meta‐Analyses (PRISMA) 2020 guidelines [23] to ensure methodological rigor and transparency in reporting. The detailed PRISMA 2020 Checklist and PRISMA 2020 Abstract Checklist have been provided as Supporting Information.

### 2.2 Inclusion and Exclusion Criteria

The following inclusion criteria were used: patients diagnosed with CRAB and studies that reported adverse outcomes, such as mortality, acute renal failure, and recurrent infections. The exclusion criteria included studies that focused on animal experiments, reviews, dissertations, and case reports, as well as those that could not be accessed or had invalid data, or duplicate publications.

### 2.3 Quality Assessment and Data Extraction

Studies that met the above criteria were tested for quality using the NOS, which had scores ranging from 0 to 9 [22]. The quality assessment was based on the following eight aspects: (1) Are the case definitions and diagnoses appropriate?, (2) Are the cases representative?, (3) How were the controls selected?, (4) Are the controls appropriately defined?, (5) Is there comparability between cases and controls?, (6) What methods were used for exposure investigation and assessment?, (7) Were the methods of investigation for cases and controls the same?, and (8) Was there a nonresponse rate?

The identified studies were screened by two investigators (J.J. and Y.H.), who then extracted data and assessed the quality of the included studies. Any discrepancies in the obtained results were discussed and resolved or arbitrated by a third investigator. Briefly, the investigators read the full text to obtain data and information, including the effect sizes (HR/OR) and 95% confidence intervals (CI) for the outcome measures.

### 2.4 Data Analysis and Statistical Methods

A meta‐analysis was conducted on the data using Stata (version 14.0 software; Stata Corp LLC, College Station, TX, USA) software. The following data were extracted including age, gender, comorbidities, disease conditions, infection sites, and procedural details. The *Q* test and *I*² test were used to test for statistical heterogeneity among the included studies. An *I*² value of < 50% indicated moderate heterogeneity, and an *I*² value of ≥ 50% reflected high heterogeneity. The fixed‐effect model was used for statistical analysis if there was no significant heterogeneity among the study results; otherwise, the random‐effects model was preferred. For all meta‐analyses performed using the random‐effects model, we report the 95% CI and the 95% prediction interval (PI). The PI estimates the range within which the true effect of a new, similar study is expected to fall, accounting for between‐study heterogeneity. The Begg and Egger tests were applied to explore the risk of publication bias. As this meta‐analysis was based entirely on previously published data, no ethical approval or patient consent was required. All analytical procedures were performed in accordance with the methodological recommendations of the Cochrane Handbook for Systematic Reviews of Interventions [24], with additional adherence to the statistical reporting guidelines proposed by Assel et al. [25] to ensure transparency and rigor in the statistical analyses.

## 3 Results

### 3.1 Flow of Included Studies

**Figure 1:**
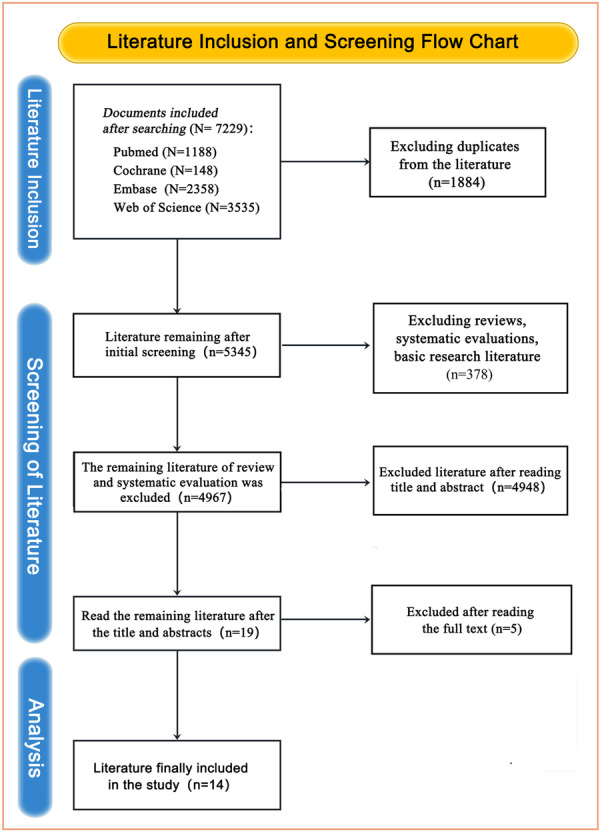
Flow diagram of included studies.

The flowchart showing the literature selection processes, such as the inclusion, screening, and analysis stages shown (Figure 1). Overall, 7229 studies were identified in the four databases (PubMed, Cochrane, Embase, and Web of Science). After removal of the duplicates, 5345 articles were retained, among which 378 articles (including reviews, systematic evaluations, and basic research) were excluded during the initial screening. The remaining 4967 articles were further screened by reading the title and abstract, and 4948 articles that did not meet the criteria were excluded. Full‐text analysis was conducted on the remaining 19 articles, and 5 articles were further excluded. Ultimately, 14 articles were included in the final meta‐analysis.

### 3.2 Study Characteristics

The basic characteristics of the 14 studies are presented in Table 1. Notably, the studies were published between 2010 and 2024, including case‐control, prospective, and retrospective studies. They were from countries, such as China (eight studies), Korea (five studies), Brazil (one study), and Italy (one study), and focused on high‐risk populations most of which were middle‐aged and elderly males (age range: 17–88 years, with mean or median ages mostly between 50 and 70 years; male ratio: 48.6%–81.1%, with males slightly more prevalent in most studies). The sample sizes varied significantly, with case groups ranging from 35 to 105 participants and control groups ranging from 20 to 207 participants. The primary outcomes reported across the studies were predominantly mortality (reported in 12 studies), followed by the incidence of acute kidney injury and the occurrence of persistent or recurrent infections beyond 72 h of tigecycline treatment. Some studies reported data variability, such as missing age or gender information for control groups. Overall, we observed the contributions of different regions in the data of severe complications, which provides essential information for subsequent meta‐analyses.

**Table 1:** Quality evaluation of the eligible studies.

No	Author	Year	Definition and diagnosis	Representativeness of cases	Selection of controls	Definition of controls	Comparability	Assessment method	Investigation method	Nonresponse rate	NOS total score
1	Dongmei Lv	2022	+	+	+	+	+	+	+	+	8
2	Shi Nae Yu	2021	+	+	+	+	+	+	+	+	8
3	Jin Woong Suh	2024	+	+	+	+	+	+	+	+	8
4	Ke Sun	2024	+	+	+	+	+	+	+	+	8
5	Chang‐Pan Liu	2016	+	+	+	+	+	+	+	+	8
6	S. Y. Kim	2012	+	+	+	+	+	+	+	+	8
7	Youn Jeong Kim	2012	+	+	+	+	_	+	+	+	7
8	Liubing Li	2024	+	+	+	+	+	+	+	+	8
9	C. G. Prates	2010	+	+	+	+	+	+	+	+	8
10	Shih‐Tse Huang	2012	+	+	+	+	−	+	+	+	7
11	Qianqian Liu	2015	+	+	+	+	+	+	+	+	8
12	Hyo‐Ju Son	2020	+	+	+	+	+	+	+	+	8
13	Shumin Gu	2023	+	+	+	+	−	+	+	+	7
14	Alessandro Russo	2019	+	+	+	+	+	+	+	+	8

*Note:* + indicates that the score is increased by one point.

### 3.3 Literature Quality Evaluation

The NOS scores of the 14 studies are shown in Table 2. Among them, 11 studies had a score of 8, reflecting high quality and full compliance with all eight evaluation criteria in the NOS scoring system. The remaining three studies scored 7, primarily due to lower scores in the comparability of cases and controls. However, their overall study designs were still relatively robust. Overall, the included studies revealed generally high‐quality and reliable data, making them ideal for subsequent analyses.

**Table 2:** General characteristics of the eligible studies.

Author	Year	Region	Number of participants		Participants' age		Male ratio (%)		Indicator
			Case group	Control	Case group	Control	Case group	Control	
Dongmei Lv [26]	2022	China	72	31	58 (17−88)	62 (27−84)	NA	NA	Mortality
Shi Nae Yu [27]	2021	Korea	103	192	68 (57−76)	70 (56−77)	67	60.4	Acute kidney injury
Jin Woong Suh [28]	2024	Korea	105	20	69 (56−77)	65 (61−74)	61.9	65	Mortality
Ke Sun [29]	2024	China	83	106	64 (54.5−74.5)	58 (50.5− 69.0)	66.3	68.9	Persistent infection/infection recurrence post‐treatment with tigecycline for 72 h
Chang‐Pan Liu [30]	2016	China	106	76	69.08 ± 16.86	69.20 ± 15.73	59.43	69.74	Mortality
S. Y. Kim [31]	2012	Korea	79	20	59.8 ± 15.7	68.3 ± 8.9	57	65	Mortality
Youn Jeong Kim [32]	2012	Korea	53	NA	59.4 ± 21.8	NA	62.3	NA	Mortality
Liubing Li [33]	2024	China	50	78	NA	NA	74	64.1	Mortality
C. G. Prates [34]	2010	Brazil	35	31	68.6 ± 15.2	71.8 ± 12.7	48.6	48.4	Mortality
Shih‐Tse Huang [35]	2012	China	56	170	69.63 ± 15.83	NA	76	NA	Mortality
Qianqian Liu [36]	2015	China	53	129	56.43 ± 18.51	50.77 ± 18.32	NA	NA	Mortality
Hyo‐Ju Son [37]	2020	Korea	90	74	62.1 ± 16.0	65.6 ± 12.7	64	72	Mortality
Shumin Gu [38]	2023	China	79	78	59 (48−73)	NA	72.6	NA	Mortality
Alessandro Russo [11]	2019	Italy	74	207	53.9 ± 17.4	63.8 ± 15.9	81.1	64.7	Mortality

*Note:* NA is not available, indicating that relevant data were not provided in the original study.

### 3.4 Subgroup Analysis of Major Risk Factors

**Figure 2:**
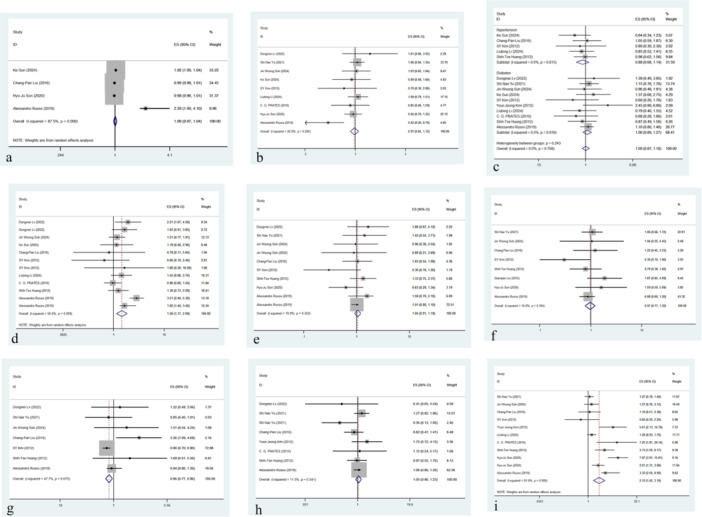
Forest map for subgroup analysis of risk factors including basic information and major comorbidities in CRAB patients. (a) Age (ES [effect size] = 1.00, 95% CI [0.97−1.04]); (b) male (ES = 0.97 [95% CI: 0.85−1.12]); (c) hypertension (ES = 0.88 [95% CI: 0.68−1.14]) and diabetes (ES = 1.06 [95% CI: 0.89−1.27]); (d) cardiovascular disease (ES = 1.56 [95% CI: 1.17−2.09]); (e) chronic kidney disease (ES = 1.04 [95% CI: 0.91−1.19]); (f) chronic lung disease (ES = 0.97 [95% CI: 0.77−1.22]); (g) chronic liver disease (ES = 0.86 [95% CI: 0.77−0.96]); (h) malignant tumor (ES = 1.05 [95% CI: 0.90−1.23]); (i) shock (ES = 2.12 [95% CI: 1.42−3.18]).

To explore the effect of major risk factors on the prognosis of patients within each subgroup, the patients were classified into nine distinct subgroups based on age, sex, comorbidities, and other relevant characteristics as presented in Figure 2. Although the composite effect value (ES, typically representing the weighted average effect size in meta‐analyses) and OR were used to examine the impact of each factor, we used the composite effect value to evaluate the overall impact of a factor [39]. This approach enabled us to integrate the research findings into a comprehensive indicator that accounts for heterogeneity and weighting across studies. To enhance the reporting of heterogeneity and the generalizability of findings, the primary results table (now presented as Table S2) has been updated to include the between‐study variance (*τ*²) and the 95% PI for every factor analyzed using the random‐effects model.

### 3.5 Age

A forest plot was developed for the four studies [11, 29, 30, 37] with significant heterogeneity (*I*² = 87.5%, *p* < 0.001), which indicated significant differences among the studies (Figure 2a). Therefore, the random‐effects model was employed in the analysis. The pooled effect size was OR [1.00, 95% CI 0.97−1.04], suggesting that age did not significantly affect the outcomes. However, related studies [40, 41, 42] revealed that age widely influenced the susceptibility to pathogen infections, and there were marked differences in susceptibility and immune responses across age groups. These differences may be due to the development and aging of the immune system and the influence of several physiological changes. Although this meta‐analysis found no significant overall effect of age on the outcomes, there was heterogeneity that may mask the variations among different age groups. In the future, more stratified age groups should be formulated to clarify the role of age in the prognosis of CRAB‐infected patients.

### 3.6 Male

Figure 2b is a forest plot including nine studies that explored the impact of gender (male) on the prognosis of CRAB patients [11, 26, 27, 28, 29, 31, 33, 34, 37]. The analysis revealed that the overall heterogeneity was low (*I*² = 20.0%, *p* = 0.265), and a fixed‐effects model was utilized for the analysis. The pooled effect size was 0.97 [95% CI: 0.85−1.12], suggesting that male gender did not markedly affect patient prognosis.

### 3.7 Hypertension and Diabetes

The forest plot analyzed the effect of a specific intervention on two groups: individuals with hypertension and those with diabetes (Figure 2c). Overall, there were five studies in the hypertension group, with low heterogeneity (*I*² = 0.0%, *p* = 0.815). A fixed‐effects model was employed for the analysis, and the pooled effect size was 0.88 [95% CI: 0.68−1.14], suggesting that the intervention did not significantly influence the patients with hypertension. For the diabetes group, 10 studies [11, 26, 27, 28, 29, 31, 32, 33, 34, 35] were included, which were found to have low heterogeneity (*I*² = 0.0%, *p* = 0.639). In the fixed‐effects model, we obtained a pooled effect size of 1.06 [95% CI: 0.89−1.27], demonstrating that the intervention did not affect outcomes in the diabetic group. Further intergroup heterogeneity analysis revealed no significant difference between the effects in the hypertension and diabetes groups (*p* = 0.243). In the overall analysis, with low heterogeneity (*I*² = 0.0%, *p* = 0.768), a pooled effect size of OR = 1.00 [95% CI: 0.87−1.16] was obtained, demonstrating that the intervention did not markedly influence the overall population.

### 3.8 Cardiovascular Disease

Figure 2d presents a forest plot comprising nine studies [11, 26, 28, 29, 30, 31, 33, 34, 35], with moderate heterogeneity (*I*² = 56.0%, *p* = 0.009). Therefore, a random‐effects model was employed for the analysis. The pooled effect size was 1.56 [95% CI: 1.17−2.09], with the 95% CI ranging from 1.17 to 2.09, and did not cross 1. This indicates that cardiovascular disease can profoundly affect the prognosis of CRAB patients. Specifically, most studies provided effect sizes within the positive range, which implies that cardiovascular disease is a predictor of poor outcomes in these patients. However, some studies indicated that the effect sizes were close to or below 1, owing to the differences in study design, sample size, and baseline characteristics among the patients. Overall, this study demonstrates that cardiovascular disease is an important risk factor predicting poor prognosis in CRAB patients, underscoring the need for more attention and formulation of strategies to manage patients with comorbid cardiovascular conditions.

### 3.9 CKD

A forest plot containing eight studies [11, 26, 27, 28, 30, 31, 35, 37] was constructed as shown in Figure 2e, with overall low heterogeneity (*I*² = 10.9%, *p* = 0.343). The analysis revealed that the results were consistent across the studies. For these studies, a fixed‐effects model was utilized, which obtained a pooled effect size of 1.04 (95% CI: 0.91−1.19), and the CI exceeded 1, suggesting no statistically significant overall effect. Notably, results of the meta‐analysis indicated that CKD did not affect the overall prognosis of CRAB patients. However, some studies [37, 43, 44] have reported that CKD patients are at higher risk of CRAB infection and mortality owing to their compromised immune systems. This is particularly observed in cases of bloodstream infection, where clinical outcomes are often worse. Altogether, these findings suggest that in specific scenarios or subgroups (e.g., patients with bloodstream infections), CKD may worsen the patient's prognosis. In the future, stratified analyses and larger‐scale studies are needed to clarify the specific effect of CKD on the prognosis of CRAB infections under varying conditions.

### 3.10 Chronic Lung Disease

A total of eight studies [11, 27, 28, 30, 31, 35, 36, 37] were included in the forest plot shown in Figure 2f, with an overall low heterogeneity (*I*² = 16.0%, *p* = 0.304), and thus were analyzed using a fixed‐effects model. The pooled effect size was 0.97 (95% CI: 0.77−1.22), with the CI exceeding 1, confirming that the pooled effect size was not statistically significant.

### 3.11 Chronic Liver Disease

In total, we included seven studies [11, 26, 27, 28, 30, 31, 35] in a forest plot (Figure 2g), which had moderate overall heterogeneity (*I*² = 47.7%, *p *= 0.075), suggesting some degree of variability among the study results, but it was not significant (Figure 2g). A fixed‐effect model was employed in the analysis. The pooled effect size was 0.86 (95% CI: 0.77−0.96), with the CI not exceeding 1, which implied that chronic liver disease can reduce the risk of adverse outcomes.

### 3.12 Malignant Tumor

Seven studies [11, 26, 27, 30, 32, 34, 35] which had low overall heterogeneity (*I*² = 11.5%, *p *= 0.341) were included in a forest plot (Figure 2h), suggesting relatively consistent results across studies. Therefore, they were analyzed using a fixed‐effect model, yielding a pooled effect size of 1.05 (95% CI: 0.90−1.23). The CI reached 1, suggesting that malignant tumors did not significantly affect the prognosis of CRAB patients.

### 3.13 Shock

A forest plot was developed to evaluate the effect of shock on the prognosis of patients with CRAB infection. A total of 10 studies [11, 27, 28, 30, 31, 32, 33, 34, 35, 37] were enrolled, with high heterogeneity (*I*² = 81.0%, *p *= 0.000), indicating significant variability among the studies (Figure 2i). Therefore, a random‐effects model was used for analysis. The pooled effect size was 2.12 (95% CI: 1.42−3.18), with the CI below 1, which indicates that shock markedly contributed to poor prognosis in CRAB patients. Overall, these results revealed that shock could predict poor prognosis in CRAB patients. However, the high heterogeneity implies that further investigations are needed to explore potential confounding factors, such as infection severity, comorbidities, or treatment strategies. Stratified analyses or subgroup studies can also clarify these differences.

### 3.14 Analysis of Other Secondary Indicators

To further determine the effect of other secondary indicators (comorbidities, surgical conditions, infection sites, and other related factors) on the prognosis of CRAB patients, OR and 95% CI were used for evaluation. The results of this analysis are presented in Table 3. It was observed that pneumonia (OR = 1.359, 95% CI: 1.107−1.668, *p* = 0.003), acute kidney injury (OR = 3.151, 95% CI: 1.687−5.883, *p* = 0.01), and hematologic malignancies (OR = 2.23, 95% CI: 1.55−3.209, *p* = 0.01) were strongly linked to poor prognosis, whereas other comorbidities, such as neurological diseases (OR = 0.79, 95% CI: 0.581−1.074, *p* = 0.133) and solid tumors (OR = 0.771, 95% CI: 0.506−1.174, *p* = 0.226), was not significantly asscociated with the prognosis. Among the surgical‐related factors, hemodialysis (OR = 1.221, 95% CI: 0.975−1.528, *p* = 0.082) was not significant but it approached the threshold of significance, whereas central venous catheterization (OR = 1.468, 95% CI: 0.801−2.691, *p* = 0.214), mechanical ventilation (OR = 1.207, 95% CI: 0.828−1.759, *p* = 0.328), and extracorporeal membrane oxygenation (ECMO) (OR = 0.75, 95% CI: 0.381−1.479, *p* = 0.407) were not significant.

**Table 3:** Analysis of other secondary indicators.

Condition	*n*	*I* ^2^ (%)	Model	OR [95% CI]			*p* value
Concomitant disease							
Neurological disease	5	57.8	Random	0.79	0.581	1.074	0.133
Cerebrovascular disease	2	44.6	Fixed	0.653	0.359	1.188	0.163
Autoimmune diseases	2	62.3	Random	1.121	0.423	2.971	0.818
Pneumonia	5	6.9	Fixed	1.359	1.107	1.668	0.003
Acute kidney injury	2	0	Fixed	3.151	1.687	5.883	0.01
Hematologic malignancy	5	0	Fixed	2.23	1.55	3.209	0.01
Solid tumor malignancy	3	0	Fixed	0.771	0.506	1.174	0.226
Operation situation							
Central venous catheter	6	59.7	Random	1.468	0.801	2.691	0.214
ECMO	2	45.3	Fixed	0.75	0.381	1.479	0.407
Mechanical ventilation	6	68.8	Random	1.207	0.828	1.759	0.328
Hemodialysis	5	6	Fixed	1.221	0.975	1.528	0.082
Central venous catheter	3	62.5	Random	1.546	0.566	4.226	0.395
Site of infection							
Catheter‐related inf	3	67.5	Random	0.961	0.594	1.556	0.873
Intra‐abdominal infe	4	54.4	Random	1.511	0.545	4.188	0.427
Urinary tract infection	2	19.9	Fixed	0.758	0.349	1.647	0.484
Skin and soft tissue	3	68.5	Random	0.805	0.231	2.801	0.733
Hospital‐acquired infection	2	0	Fixed	1.03	0.862	1.232	0.742
Other indicators							
Transplantation	3	0	Fixed	2.247	0.924	5.46	0.074
ICU admission	9	83	Random	1.359	0.991	1.862	0.057
Recent surgery	4	69.6	Random	1.573	0.914	2.707	0.102
Neutropenia, *n* (%)	5	32.2	Fixed	2.737	1.479	5.063	0.001
Immunosuppressive agents	3	56.3	Random	1.34	0.704	2.549	0.373
Thrombocytopenia	2	0	Fixed	1.488	1.097	2.019	0.011
Immunosuppressive steroids	3	80.2	Random	1.38	0.53	3.59	0.509
Primary bacteremia	3	72.4	Random	1.332	0.461	3.844	0.596
Steroid therapy	4	0	Fixed	1.288	1.013	1.636	0.039

Analysis of infection sites found no significant correlation between catheter‐related infections (OR = 0.961, 95% CI: 0.594−1.556, *p* = 0.873), intra‐abdominal infections (OR = 1.511, 95% CI: 0.545−4.188, *p* = 0.427), or urinary tract infections (OR = 0.758, 95% CI: 0.349−1.647, *p* = 0.484), with poor prognosis. Although intra‐abdominal infections indicated a potential trend of increased risk. Among other factors, neutropenia (OR = 2.737, 95% CI: 1.479−5.063, *p* = 0.001), thrombocytopenia (OR = 1.488, 95% CI: 1.097−2.019, *p* = 0.011), and steroid therapy (OR = 1.288, 95% CI: 1.013−1.636, *p* = 0.039) significantly increased the risk of poor prognosis, while ICU admission (OR = 1.359, 95% CI: 0.991−1.862, *p* = 0.057) and recent surgery (OR = 1.573, 95% CI: 0.914−2.707, *p* = 0.102) indicated potential risks, but were not significant. Key prognostic factors identified among comorbidities included acute organ injury and hematological abnormalities. Conversely, the influence of surgical interventions and infection‐related variables remains uncertain and requires additional research for clarification. Although the *p* values did not indicate statistical significance (all *p* > 0.05), the direction of the pooled estimates suggests a potential trend toward increased risk for intra‐abdominal infections, which may warrant further investigation in larger cohorts.

### 3.15 Predictors of Patient Death

To clarify the risk factors associated with mortality in CRAB patients, predictive factors from 12 studies focusing on death as the adverse outcome were analyzed. These factors included basic information, comorbidities, sites of infection, surgical conditions, and other risk indicators, and their results are shown in Table 4. It was observed that cardiovascular disease (OR = 1.592, 95% CI: 1.217−2.081), hematologic malignancy (OR = 2.23, 95% CI: 1.55−3.209), pneumonia (OR = 1.276, 95% CI: 1.017−1.601), shock (OR = 2.331, 95% CI: 1.497−3.63), neutropenia (OR = 2.737, 95% CI: 1.479−5.063), and thrombocytopenia (OR = 1.488, 95% CI: 1.097−2.019) significantly increased the risk of mortality. Furthermore, intra‐abdominal infection (OR = 1.511, 95% CI: 0.545−4.188) and recent surgery (OR = 1.573, 95% CI: 0.914−2.707) were potential risks, although not statistically significant. In contrast, factors such as gender, age, certain infection sites (e.g., urinary tract infections), and specific treatment methods (e.g., mechanical ventilation and ECMO) were not significantly associated with mortality [45]. In summary, the analysis identifies cardiovascular disease, hematologic malignancies, shock, and hematological abnormalities as key predictors of mortality in patients with CRAB infection. These findings emphasize the importance of targeted clinical management for high‐risk individuals to help reduce mortality rates.

**Table 4:** Predictors of patient death.

Condition	*n*	*I* ^2^ (%)	Model	OR [95% CI]			*p* value
Basic information							
Male	7	34.4	Fixed	0.936	0.788	1.111	0.448
Age	3	88.3	Random	1.003	0.946	1.063	0.926
Concomitant disease							
Cardiovascular disease	11	59.1	Random	1.592	1.17	2.167	0.003
Chronic liver disease	6	56.4	Random	1.051	0.794	1.392	0.726
Chronic kidney disease	9	17.4	Fixed	1.034	0.902	1.186	0.632
Chronic lung disease	7	26.3	Fixed	0.944	0.728	1.224	0.665
Diabetes	8	0	Fixed	1.02	0.832	1.25	0.851
Hypertension	4	0	Fixed	0.94	0.709	1.245	0.664
Malignancy	7	15.4	Fixed	1.021	0.859	1.212	0.817
Hematologic malignancy	5	0	Fixed	2.23	1.55	3.209	0
Solid tumor malignancy	3	0	Fixed	0.771	0.506	1.174	0.226
Neurological disease	3	0	Fixed	0.995	0.828	1.197	0.959
Cerebrovascular disease	2	44.6	Fixed	0.653	0.359	1.188	0.163
Autoimmune diseases	2	62.3	Random	1.121	0.423	2.971	0.818
Acute kidney injury	2	0	Fixed	3.151	1.687	5.883	0
Site of infection							
Catheter‐related infection	3	67.5	Random	0.961	0.594	1.556	0.873
Intra‐abdominal infection	4	54.4	Random	1.511	0.545	4.188	0.427
Urinary tract infection	2	19.9	Fixed	0.758	0.349	1.647	0.484
Skin and soft tissue	3	68.5	Random	0.805	0.231	2.801	0.733
Hospital‐acquired infection	2	0	Fixed	1.03	0.862	1.232	0.742
Operation situation							
Pneumonia	4	0	Fixed	1.276	1.017	1.6	0.035
Mechanical ventilation	6	68.8	Random	1.207	0.828	1.759	0.328
Hemodialysis	5	6	Fixed	1.221	0.975	1.528	0.082
ECMO, *n* (%)	2	45.3	Fixed	0.75	0.381	1.479	0.407
Central venous catheter	3	62.5	Random	1.546	0.566	4.226	0.395
Other indicators							
Transplantation	3	0	Fixed	2.247	0.924	5.46	0.074
Shock	10	79.2	Random	2.331	1.497	3.63	0
ICU admission	9	83	Random	1.359	0.991	1.862	0.057
Recent surgery	3	79.6	Random	1.48	0.713	3.072	0.293
Neutropenia	5	32.2	Fixed	2.737	1.479	5.063	0.001
Immunosuppressive agents	2	40.8	Fixed	0.984	0.568	1.705	0.954
Thrombocytopenia	2	0	Fixed	1.488	1.097	2.019	0.011
Immunosuppressive steroids	3	80.2	Random	1.38	0.53	3.59	0.509
Primary bacteremia	3	72.4	Random	1.332	0.461	3.844	0.596
Steroid therapy	4	0	Fixed	1.288	1.013	1.636	0.039

### 3.16 Offset Check

To investigate the existence of systematic bias in the results and determine the reliability and robustness of the analyses, we first generated funnel plots for major comparison (Figure S1) and then performed Begg's test and Egger's test on indicators reported by at least three studies (Table 5). The finding showed that there was no marked bias in both Begg's test and Egger's test (*p* > 0.05), such as age (Begg's *p* = 0.497, Egger's *p* = 0.235), cardiovascular disease (Begg's *p* = 0.317, Egger's *p* = 0.442), and mechanical ventilation (Begg's *p* = 0.196, Egger's *p* = 0.661). However, a few indicators, such as hematologic malignancy (Begg's *p* = 0.317, Egger's *p* = 0.045), steroid therapy (Begg's *p* = 0.309, Egger's *p* = 0.057), neutropenia (Begg's *p* = 0.142, Egger's *p* = 0.045), and transplantation (Begg's *p* = 0.117, Egger's *p* = 0.003), exhibited potential bias in Egger's test, which indicated the presence of slight systematic errors in the associated studies. It should be noted that the visual interpretation of these funnel plots for publication bias may be limited when the number of included studies is fewer than 10. Overall, the bias tests revealed that most indicators exhibited robust results, but a few showed publication bias or data heterogeneity.

**Table 5:** Offset check.

Indicator	*n*	Begg's		Egger's	
		*z*	*p*	Bias	*p*
Age	4	0.68	0.497	3.69	0.235
Cardiovascular disease	12	−0.27	0.784	−1.13	0.307
Catheter‐related infection	3	−1.57	0.117	−2.84	0.316
Central venous catheter	3	0.52	0.602	0.33	0.913
Chronic kidney disease	10	−1.34	0.18	−0.01	0.98
Chronic liver disease	7	1.35	0.176	1.48	0.029
Chronic lung disease	8	0.49	0.621	0.17	0.858
Diabetes	10	−0.27	0.788	−0.21	0.76
Hematologic malignancy	5	0.98	0.327	1.03	0.214
Hemodialysis	5	0.49	0.624	2.59	0.242
Hypertension	5	−0.98	0.327	−0.98	0.486
ICU admission	9	1.88	0.061	4.26	0.11
Immunosuppressive agent use	3	−0.52	0.602	−0.15	0.989
Immunosuppressive status	3	0.52	0.602	2.11	0.385
Intra‐abdominal infection	4	−0.68	0.497	−3.28	0.133
Male	9	−1.67	0.095	−0.91	0.32
Malignancy	8	−0.74	0.458	−0.63	0.352
Mechanical ventilation	6	0.19	0.851	1.46	0.503
Neurological disease	5	0	1	−1.72	0.313
Neutropenia	5	−1.47	0.142	−1.28	0.273
Pneumonia	5	0	1	0.03	0.981
Primary bacteremia	3	0.52	0.602	3.68	0.459
Recent surgery	4	−0.68	0.497	−5.08	0.395
Shock	11	1.48	0.139	2.87	0.078
Skin and soft tissue infection	3	−0.52	0.602	−3.33	0.775
Solid tumor malignancy	3	1.57	0.117	2.48	0.113
Steroid therapy	4	2.04	0.042	3.78	0.026
Transplantation	3	−1.57	0.117	−7.75	0.045

## 4 Discussion

CRAB is a pathogen that poses a significant public health threat, owing to its high mortality rates, lack of effective treatments, and rapid nosocomial transmission [46, 47]. Here, we performed a systematic literature review and meta‐analysis to identify the adverse outcomes of patients with CRAB infections and their associated predictors. This will help optimize clinical management, develop personalized treatment plans, and improve patient prognosis.

The analysis revealed that age affected the risk of CRAB infection and immune responses [48, 49], but the meta‐analysis found no effects of age on the prognosis of CRAB infection (ES = 1.00, 95% CI: 0.97−1.04). This result may be explained by the possibility that the age distribution was concentrated within a specific range (age range: 17–88 years, with mean or median ages ranging between 50 and 70 years), indicating that the sample size in the included studies was insufficient to detect the influence of age on prognosis. Further stratified analyses and large‐scale studies are warranted to clarify the effect of age on the prognosis of CRAB patients. In the gender analysis (ES = 0.97, 95% CI: 0.85−1.12), we observed that males had no effect on the prognosis of CRAB patients. Although gender may influence immune responses and prognoses in certain infectious diseases [50], the prognosis of CRAB patients is generally affected by multiple factors, such as underlying diseases, infection sites, treatment timing, and severity. Gender may play a secondary role relative to the more critical prognostic determinants; therefore, gender differences may not significantly affect overall prognosis.

Regarding comorbidities, the results indicated that hypertension (ES = 0.88, 95% CI: 0.68−1.14) and diabetes (ES = 1.06, 95% CI: 0.89−1.27) did not significantly affect the patient's prognosis. Hypertension and diabetes are common chronic conditions, which are uniformly prevalent in the study population and lacked significant individual effect on the prognosis of CRAB‐infected patients. However, other studies [51] through Cox multivariate analysis of CRAB‐infected patients have demonstrated that diabetic patients have an increased mortality risk. Another study reported that hyperglycemia (even with mild elevation) correlated with high mortality in critically ill patients, regardless of disease severity [52]. This may be associated with oxidative stress and pro‐inflammatory responses which are common in hyperglycemic patients, which impairs the immune function, but the exact mechanisms need to be further clarified. In contrast, cardiovascular diseases (ES = 1.56, 95% CI: 1.17−2.09) and hematologic malignancies (ES = 2.23, 95% CI: 1.55−3.209) were correlated with increased risk of adverse outcomes. The former can induce systemic inflammation and organ dysfunction [53, 54], while the latter causes immunosuppression and treatment‐associated complications [55]. Previously, carbapenem‐resistant bacteria were reported to induce a BSI mortality rate of 45%−80% in hematologic malignancy patients [56]. This suggests that the type of comorbidity may influence the prognosis of CRAB patients.

In the analysis of acute complications, acute kidney injury (OR = 3.151, 95% CI: 1.687−5.883), neutropenia (OR = 2.737, 95% CI: 1.479−5.063), and thrombocytopenia (OR = 1.488, 95% CI: 1.097−2.019) predicted poor prognosis. Multiple studies [57, 58] have also demonstrated that acute kidney injury is a risk factor for increased mortality in MDR patients. Data from a study from Seoul National University Children's Hospital [59] indicated that neutropenia could independently increase the risk of mortality in patients with *Acinetobacter bacteremia*. Many other investigations have reported a significant association between thrombocytopenia and mortality in the infected patients [60, 61]. A recent 6‐year study in China obtained that a platelet count < 50 × 10^9^/L increased the 30‐day mortality risk in infected patients by nine times [62]. These acute complications may elevate the mortality risk by weakening the patient's immune defense or exacerbating systemic inflammation. Moreover, shock (OR = 2.12, 95% CI: 1.42−3.18) can increase the likelihood of adverse outcomes, further emphasizing the importance of early recognition and intervention in shock states in CRAB‐infected patients.

From an etiologic perspective, pneumonia (OR = 1.359, 95% CI: 1.107−1.668) was significantly correlated with poor outcomes, particularly in the context of ventilator‐associated pneumonia attributed to drug‐resistant Acinetobacter, which has been strongly implicated in the high mortality rates seen in critically ill patients [63, 64, 65]. Conversely, catheter‐related infections (OR = 0.961, 95% CI: 0.594−1.556) and urinary tract infections (OR = 0.758, 95% CI: 0.349−1.647) did not influence the mortality risk, a finding that appears to deviate from the outcomes of prior research [66] and needs to be further clarified. In terms of therapeutic factors, interventions such as mechanical ventilation, central venous catheter insertion, and ECMO appeared to have a limited direct impact on patient prognosis. However, these interventions may be intricately linked to the severity of the primary diseases, hence warranting further investigation to elucidate their potential influences.

While this meta‐analysis has focused on clinical prognostic indicators, it is imperative to note that the antimicrobial resistance profile of the infecting CRAB strain is a critical underlying determinant of outcome [67, 68]. Differences in susceptibility to last‐line agents, such as colistin and tigecycline, directly influence treatment efficacy and were not uniformly reported or adjusted for in the included studies. The clinical factors identified here (e.g., shock, hematologic malignancy) likely exert their effect within the constrained therapeutic landscape created by carbapenem resistance. As most included studies were conducted in China and Korea, the generalizability of our findings may be limited. Nonetheless, the identified predictors—such as cardiovascular disease, shock, acute kidney injury, and hematologic malignancy—are consistent with results from other regions, suggesting these associations are driven by fundamental pathophysiological mechanisms rather than regional factors. Future prospective studies that integrate detailed molecular resistance data with clinical variables are needed to build even more robust predictive models.

### 4.1 Limitations

This study has several limitations. (1) Demographic data such as age and gender were incomplete in some studies, and variations existed in the definitions of variables (e.g., age categories, septic shock). Potential publication bias or systematic error cannot be excluded for certain indicators such as hematologic malignancy and neutropenia. Future well‐designed multicenter studies are needed to validate these findings and strengthen the evidence for the clinical management of CRAB infections. (2) Furthermore, in our meta‐analysis, we selected between fixed‐effect and random‐effects models based on the *I*² statistic. While this is a conventional approach to balance specificity and generalizability, we acknowledge the theoretical argument for the default use of random‐effects models in the presence of any clinical diversity, regardless of the statistical measure of heterogeneity. (3) Due to limited study numbers and incomplete reporting of covariates (e.g., gender ratio, age, clinical features), subgroup and meta‐regression analyses could not be performed. Future studies with standardized reporting will enable a more detailed exploration of heterogeneity. (4) Interventions such as mechanical ventilation and ECMO likely reflect underlying disease severity rather than act as independent prognostic factors. Furthermore, antimicrobial resistance patterns were not uniformly reported, yet they remain crucial determinants of treatment outcomes. Future prospective studies integrating clinical and microbiological data are needed to refine prognostic assessment in CRAB infections.

## Author Contributions

Conceptualization: Ling Yan Du and Jie Jin. Methodology: Jie Jin. Software: Yan Hu. Validation: Jie Jin and Yan Hu. Formal analysis: Jie Jin and Yan Hu. Data curation: Jie Jin and Yan Hu. Writing the original draft preparation: Jie Jin and Yan Hu. Writing, review, and editing: Ling Yan Du. Visualization: Jie Jin. Project administration: Ling Yan Du. All authors have read and agreed to the published version of the manuscript. All authors have read and approved the final version of the manuscript.

## Funding

The authors received no specific funding for this work.

## Ethics Statement

The authors have nothing to report.

## Consent

The authors have nothing to report.

## Conflicts of Interest

The authors declare no conflicts of interest.

## Transparency Statement

The lead author Ling Yan Du affirms that this manuscript is an honest, accurate, and transparent account of the study being reported; that no important aspects of the study have been omitted; and that any discrepancies from the study as planned (and, if relevant, registered) have been explained.

## Supporting information


**Figure S1:** Funnel Plots for Publication Bias Assessment of Nine Risk Factors. **Table S1:** Computer search of PubMed, Embase and The Cochrane Library databases was conducted before December 2024. **Table S2:** Random‐Effects Meta‐Analysis of Prognostic Factors for Adverse Outcomes in CRAB Infection, Reporting τ^2^ and 95% PI.

PRISMA_2020_checklist_HSR_2025_03_1115.

## Data Availability

The data supporting the findings of this study are available within the article and its supplementary materials. The corresponding author, Linyan Du, had full access to all of the data and takes complete responsibility for the integrity of the data and accuracy of the data analysis.
