# Supplementary material for: Prognostic Indicators of Carbapenem‐Resistant Acinetobacter baumannii Infection: A Meta‐Analysis and Systematic Review
Source: Health Sci Rep. 2025 Dec 14;8(12):e71639. doi: 10.1002/hsr2.71639 (PMC12703131; doi:10.1002/hsr2.71639)
Supplement: Supplementary file 1 — Figure S1: Funnel Plots for Publication Bias Assessment of Nine Risk Factors. Table S1: Computer search of PubMed, Embase and The Cochrane Library databases was conducted before December 2024. Table S2: Random‐Effects Meta‐Analysis of Prognostic Factors for Adverse Outcomes in CRAB Infection, Reporting τ2 and 95% PI. [file HSR2-8-e71639-s001.docx]

**Prognostic indicators of carbapenem-resistant *Acinetobacter baumannii* infection: A meta-analysis and systematic review**

**Supplementary material**

Table S1. Computer search of PubMed, Embase and The Cochrane Library databases was conducted before December 2024. English search terms include Resistance, Multiple Drug Resistance, Acinetobacter baumannii, Death, etc. PubMed is taken as an example. For specific search strategies, see Table S1 in the annex.

| Database | Search Strategy |
| --- | --- |
| PubMed | ((((((((((((((((((Resistance[Title/Abstract]) OR (Resistance*[Title/Abstract])) OR (resistant*[Title/Abstract])) OR (Drug resistance[Title/Abstract])) OR (drug-fast[Title/Abstract])) OR (Multiple Drug Resistance[Title/Abstract])) OR (Resistance, Multiple Drug[Title/Abstract])) OR (Multi-Drug Resistance[Title/Abstract])) OR (Multidrug Resistance[Title/Abstract])) OR (Extensively Drug Resistance[Title/Abstract])) OR (Extensively Drug Resistances[Title/Abstract])) OR (Resistance, Extensively Drug[Title/Abstract])) OR (Drug Resistance, Extensively[Title/Abstract])) OR (Extensively-Drug Resistance[Title/Abstract])) OR (Extensively-Drug Resistances[Title/Abstract])) OR (Resistance, Extensively-Drug[Title/Abstract])) AND ((((((Carbapenem[Title/Abstract]) OR (Carbapenem*[Title/Abstract])) OR (Carbapenems[Title/Abstract])) OR (Carbapenem[Title/Abstract])) OR (Antibiotics, Carbapenem[Title/Abstract])) OR (Carbapenem Antibiotics[Title/Abstract]))) AND ((((((((Acinetobacter baumannii[Title/Abstract]) OR (Acinetobacter baumannii*[Title/Abstract])) OR (Bacterium anitratum[Title/Abstract])) OR (Hospital-acquired infections[Title/Abstract])) OR (Severe infections[Title/Abstract])) OR (Bloodstream infection[Title/Abstract])) OR (Urinary tract infection[Title/Abstract])) OR (Wound infections[Title/Abstract]))) AND (((((((((((((((((Prognosis[Title/Abstract]) OR (Prognostic*[Title/Abstract])) OR (Forecast[Title/Abstract])) OR (Risk factor[Title/Abstract])) OR (Death[Title/Abstract])) OR (hospitalized[Title/Abstract])) OR (readmission[Title/Abstract])) OR (Disease outcome[Title/Abstract])) OR (Survival rate[Title/Abstract])) OR (Survival[Title/Abstract])) OR (Cure rate[Title/Abstract])) OR (Cure[Title/Abstract])) OR (Disease resolution[Title/Abstract])) OR (Risk assessment[Title/Abstract])) OR (Prognostic assessment[Title/Abstract])) OR (Prognostic models[Title/Abstract])) OR (Prediction models[Title/Abstract])) |

Table S2. Random-Effects Meta-Analysis of Prognostic Factors for Adverse Outcomes in CRAB Infection, Reporting τ² and 95% PI.

| **Risk Factor** | **No. of Studies (N)** | **OR (95% CI)** | **I² (%)** | **τ²** | **95% Prediction Interval** |
| --- | --- | --- | --- | --- | --- |
| ****Demographic**** |  |  |  |  |  |
| Age | 3 | 1.003 (0.946-1.063) | 88.3 | 0.007 | 0.877 - 1.146 |
| ****Comorbidities**** |  |  |  |  |  |
| Cardiovascular disease | 11 | 1.592 (1.170-2.167) | 59.1 | 0.036 | 0.947 - 2.675 |
| Chronic liver disease | 6 | 1.051 (0.794-1.392) | 56.4 | 0.027 | 0.646 - 1.714 |
| Autoimmune diseases | 2 | 1.121 (0.423-2.971) | 62.3 | 0.364 | 0.197 - 6.390 |
| ****Infection Site**** |  |  |  |  |  |
| Catheter-related infection | 3 | 0.961 (0.594-1.556) | 67.5 | 0.124 | 0.395 - 2.339 |
| Intra-abdominal infection | 4 | 1.511 (0.545-4.188) | 54.4 | 0.308 | 0.425 - 5.390 |
| Skin and soft tissue infection | 3 | 0.805 (0.231-2.801) | 68.5 | 0.535 | 0.109 - 5.940 |
| ****Interventions & Conditions**** |  |  |  |  |  |
| Mechanical ventilation | 6 | 1.207 (0.828-1.759) | 68.8 | 0.062 | 0.606 - 2.404 |
| Central venous catheter | 3 | 1.546 (0.566-4.226) | 62.5 | 0.363 | 0.273 - 8.770 |
| Transplantation | 3 | 2.247 (0.924-5.460) | 0.0 | 0.000 | 0.928 - 5.440 |
| Shock | 10 | 2.331 (1.497-3.630) | 79.2 | 0.203 | 0.878 - 6.180 |
| ICU admission | 9 | 1.359 (0.991-1.862) | 83.0 | 0.181 | 0.573 - 3.220 |
| Recent surgery | 3 | 1.480 (0.713-3.072) | 79.6 | 0.581 | 0.239 - 9.160 |
| Immunosuppressive steroids | 3 | 1.380 (0.530-3.590) | 80.2 | 0.648 | 0.196 - 9.710 |
| Primary bacteremia | 3 | 1.332 (0.461-3.844) | 72.4 | 0.384 | 0.213 - 8.340 |

****Note:**** All analyses were performed using a random-effects model; τ², between-study variance..


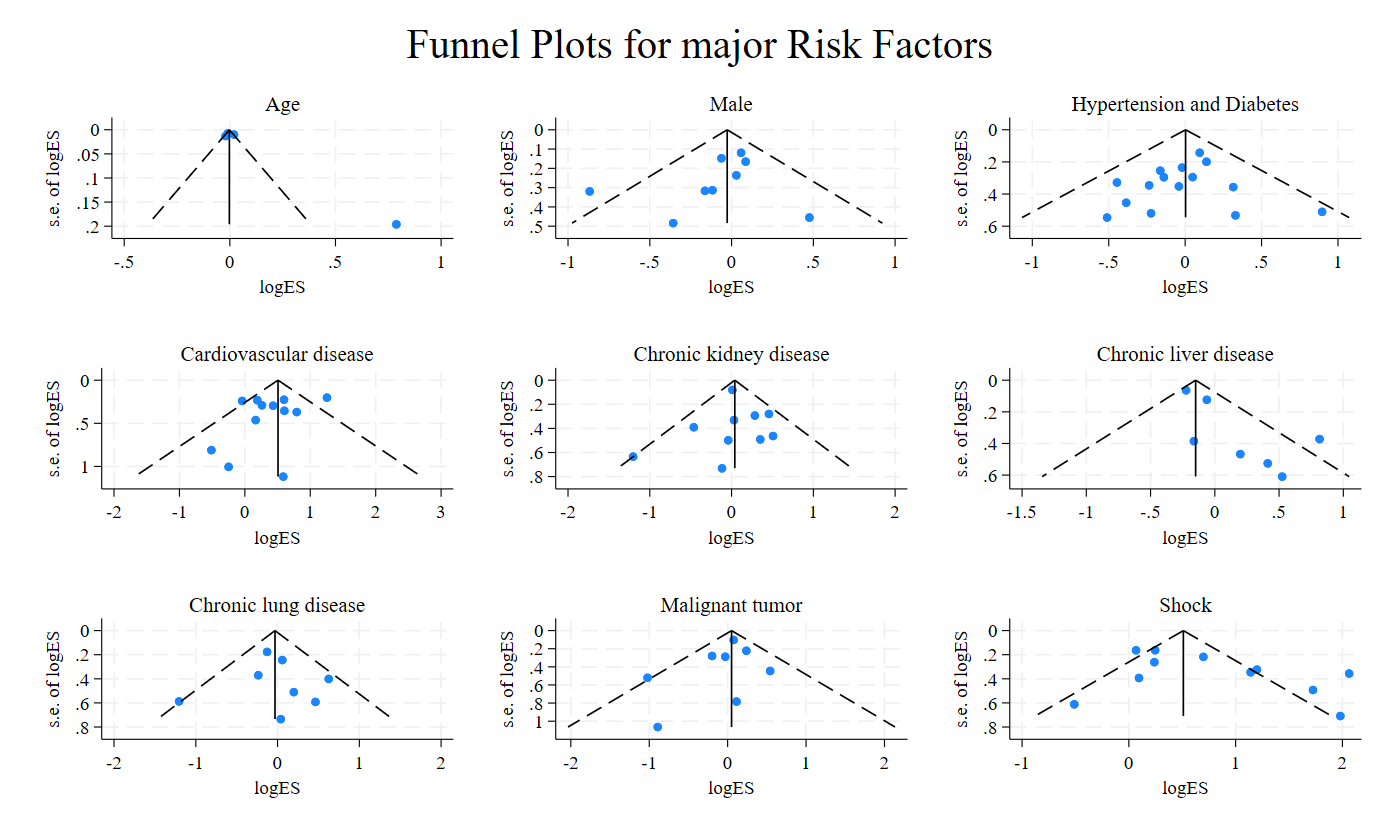
Figure S1. Funnel Plots for Publication Bias Assessment of Nine Risk Factors. Funnel plots evaluating publication bias across nine risk factors. Symmetrical distribution of studies around the null value (logES = 0) was observed for most factors including male, hypertension and diabetes, chronic lung disease, and malignant tumor, suggesting minimal publication bias. Moderate asymmetry was noted for cardiovascular disease and shock, indicating potential publication bias or clinical heterogeneity. Age, chronic kidney disease, and chronic liver disease showed generally symmetrical patterns with acceptable reporting balance.
